# The role of PPARγ in carbon nanotube-elicited granulomatous lung inflammation

**DOI:** 10.1186/1465-9921-14-7

**Published:** 2013-01-23

**Authors:** Isham Huizar, Anagha Malur, Janki Patel, Matthew McPeek, Larry Dobbs, Christopher Wingard, Barbara P Barna, Mary Jane Thomassen

**Affiliations:** 1 Department of Internal Medicine, Division of Pulmonary and Critical Care Medicine, The Brody School of Medicine, East Carolina University, 3E-149 Brody Medical Sciences Building, Greenville, NC, 27834, USA; 2 Department of Pathology, East Carolina University, Greenville, USA; 3 Department of Physiology, East Carolina University, Greenville, USA; 4 Department of Microbiology and Immunology, East Carolina University, Greenville, USA

## Abstract

**Background:**

Although granulomatous inflammation is a central feature of many disease processes, cellular mechanisms of granuloma formation and persistence are poorly understood. Carbon nanoparticles, which can be products of manufacture or the environment, have been associated with granulomatous disease. This paper utilizes a previously described carbon nanoparticle granuloma model to address the issue of whether peroxisome proliferator-activated receptor gamma (PPARγ), a nuclear transcription factor and negative regulator of inflammatory cytokines might play a role in granulomatous lung disease. PPARγ is constitutively expressed in alveolar macrophages from healthy individuals but is depressed in alveolar macrophages of patients with sarcoidosis, a prototypical granulomatous disease. Our previous study of macrophage-specific PPARγ KO mice had revealed an intrinsically inflammatory pulmonary environment with an elevated pro-inflammatory cytokines profile as compared to wild-type mice. Based on such observations we hypothesized that PPARγ expression would be repressed in alveolar macrophages from animals bearing granulomas induced by MWCNT instillation.

**Methods:**

Wild-type C57Bl/6 and macrophage-specific PPARγ KO mice received oropharyngeal instillations of multiwall carbon nanotubes (MWCNT) (100 μg). Bronchoalveolar lavage (BAL) cells, BAL fluids, and lung tissues were obtained 60 days post-instillation for analysis of granuloma histology and pro-inflammatory cytokines (osteopontin, CCL2, and interferon gamma [IFN-γ] mRNA and protein expression.

**Results:**

In wild-type mice, alveolar macrophage PPARγ expression and activity were significantly reduced in granuloma-bearing animals 60 days after MWCNT instillation. In macrophage-specific PPARγ KO mice, granuloma formation was more extensive than in wild-type at 60 days after MWCNT instillation. PPARγ KO mice also demonstrated elevated pro-inflammatory cytokine expression in lung tissue, laser-microdissected lung granulomas, and BAL cells/fluids, at 60 days post MWCNT exposure.

**Conclusions:**

Overall, data indicate that PPARγ deficiency promotes inflammation and granuloma formation, suggesting that PPARγ functions as a negative regulator of chronic granulomatous inflammation.

## Background

Investment in nanotechnology is currently estimated to constitute approximately 18 billion dollars nationally, with commercial products ranging from sunscreens to bicycle frames [1]. While the environmental and occupational health impacts of nanotechnology remain to be established, evidence of toxicity has emerged from some experimental models where carbon-based nanomaterials persist for long periods in lung tissue and induce granulomatous changes (reviewed in [2,3]. Granulomatous disease may occur in human lung in response to a wide spectrum of environmental stimuli including intracellular pathogens, inert materials, and organic antigens. In sarcoidosis, a prototypical granulomatous disease, the etiology remains obscure [4]. Multiple occupational and environmental risk factors have been linked to sarcoidosis, including exposure to particulates from wood-burning stoves, fireplaces, firefighting, and the World Trade Center disaster – conditions that favor carbon nanotube formation in ambient air [5-8].

Granulomatous changes have been reported in association with instillation of single wall carbon nanotubes [9-11]. We recently reported a novel murine model of chronic granulomatous inflammation elicited by exposure to multiwall carbon nanotubes (MWCNT) [12]. This model demonstrated several key similarities with granulomas encountered in human sarcoidosis: (a) chronicity with persistence up to 90 days; (b) macrophage and T cell recruitment; and (c) marked elevation of inflammatory cytokines [12]. Previously published murine granuloma models utilized sepharose beads to elicit acute granulomas that formed and resolved within three weeks [13].

The transcription factor, PPARγ, is a critical regulator of lipid and glucose metabolism but also recognized as a negative regulator of genes linked to inflammatory events [14]. Alveolar macrophages of healthy individuals constitutively express PPARγ but PPARγ is deficient in alveolar macrophages from patients with severe sarcoidosis, suggesting that this factor represents an important regulator of inflammation [15]. Based on these observations we hypothesized that PPARγ might play a role in the formation of MWCNT granulomas. To address this hypothesis we first examined the effects of MWCNT instillation on PPARγ expression and activity in wild-type mice. Secondly, we investigated the effect of pre-existing PPARγ deficiency on MWCNT-elicited granulomas by utilizing macrophage-specific PPARγ KO mice. Results suggested that PPARγ functions as a negative regulator of granuloma formation in response to MWCNT instillation.

## Methods

All studies were conducted in conformity with Public Health Service (PHS) Policy on humane care and use of laboratory animals and were approved by the institutional animal care committee.

### Mice

C57BL/6J wild-type mice from Jackson Laboratories and macrophage-specific PPARγ KO conditional mice as previously described [16] were utilized in experiments as indicated.

### Characterization of carbon nanotubes

MWCNTs (catalogue number 900–1501, lot GS1801), grown via chemical vapor deposition, were obtained from SES Research (Houston, TX). We determined the structure of MWCNTs by scanning electron microscopy. Nitrogen adsorption studies were performed using a physisorption analyzer (ASAP 2010; Micromeritics, Norcross, GA). The details of the MWCNT characterization were previously described by Huizar et al. [12].

### Instillation of carbon nanotubes

Procedures were performed according to the East Carolina University Office of Environmental Health and Safety. An oropharyngeal instillation was performed after sedation with isofluorane. Sixty days after sham treatment (PBS/35% surfactant) or exposure to 100 ug MWCNT, the mice were sacrificed, and bronchoalveolar lavage (BAL) or lungs were harvested for further analysis as previously described [12].

### Histological analysis

Lungs were dissected and fixed in PBS-buffered 4% formaldehyde. A semiquantitative scoring system was devised to allow for a relative comparison of the numbers and quality of the granulomas formed in the C57 and PPARγ mice strains. The glass slides with the Hematoxylin and Eosin stained sections of lung from each of the experimental mice was assigned a score of between 0 and 5 by two independent investigators using the following scoring system: (score 0) – no granulomas or aggregates of macrophages seen; (score 1) - few small groups of macrophages but no well-formed granulomas; (score 2) - scattered small granulomas not easily seen on scanning power (20X); (score 3) - scattered small granulomas easily seen on scanning power (20X); (score 4) - scattered small granulomas with occasional larger granulomas seen on scanning power (20X); (score 5) - numerous large granulomas easily seen on scanning power. The scores obtained by the two investigators were averaged for the final analysis.

### Bronchoalveolar Lavage (BAL)

BAL cells were obtained as previously described [16]. Differential cell counts were determined from cytospins stained with a modified Wright’s stain. Mean viability of lavage cells was greater than 95% as determined by trypan blue dye exclusion.

### RNA purification and analysis

Total RNA was extracted from total BAL cells by RNeasy protocol (Qiagen, Valencia, CA). Expression of mRNA was determined by real time qPCR using the ABI Prism 7300 Detection System (TaqMan; Applied Biosystems, Foster City, CA) according to the manufacturer’s instructions. RNA specimens were analyzed in duplicate using primer sets for IFN-γ, osteopontin, and CCL2 (MCP-1). Threshold cycle (CT) values for genes of interest were normalized to a housekeeping gene glyceraldehyde 3 phosphate dehydrogenase, (GAPDH) as previously described [16]. Data were expressed as a fold change in mRNA expression of MWCNT-bearing mice relative to sham control values.

### Immunocytochemistry

Immunocytochemistry was performed on cytospin preparations from freshly isolated C57Bl/6 alveolar macrophages for basal expression levels of PPARγ (Santa Cruz Biotech, Santa Cruz, CA). Slides were fixed with 4% paraformaldehyde–PBS, then permeabilized with Triton X-100 and stained with anti-PPARγ antibody at 1:500 dilution, followed by Alexa conjugated goat anti rabbit IgG (Invitrogen). Staining of lung tissues with antibody to IFN-γ, CD3 and MOMA2 (anti-monocyte and macrophage) was carried out as described previously [16]. Slides were counter stained with Propidium Iodide (PI) [Vector Laboratories] or DAPI (Invitrogen) to facilitate nuclear localization.

### Laser-capture microdissection

Lung tissues from sham-treated mice (controls) and both granulomatous, and non-granulomatous lung tissues from MWCNT-instilled mice were dissected using a Zeiss PALM IV LCM (Carl Zeiss MicroImaging LLC, Thornwood, New York) system. Frozen sections of the lung were processed as previously described [12]. RNA was extracted from tissue sections, converted into cDNA or preamplified, as described previously [12].

### Quantitation of cytokine proteins

Murine osteopontin was quantified in BAL fluids using an ELISA assay (R&D stystems, Minneapolis, MN), as previously described [12]. Murine IFN-γ and CCL2 (MCP-1) were quantified by electrochemiluminescence detection (Meso Scale Diagnostics, Gaithersburg, MD).

### Statistical analyses

Data were analyzed by student’s t-test or Mann–Whitney using Prism software (GraphPad, Inc., San Diego, CA.). Values from treated were compared to sham treated animals. Data are expressed as mean ± SEM. Significance was defined as p ≤ 0.05.

## Results

### PPARγ expression and activity are decreased in alveolar macrophages from MWCNT-instilled wild-type C57Bl/6 mice

**Figure 1:**
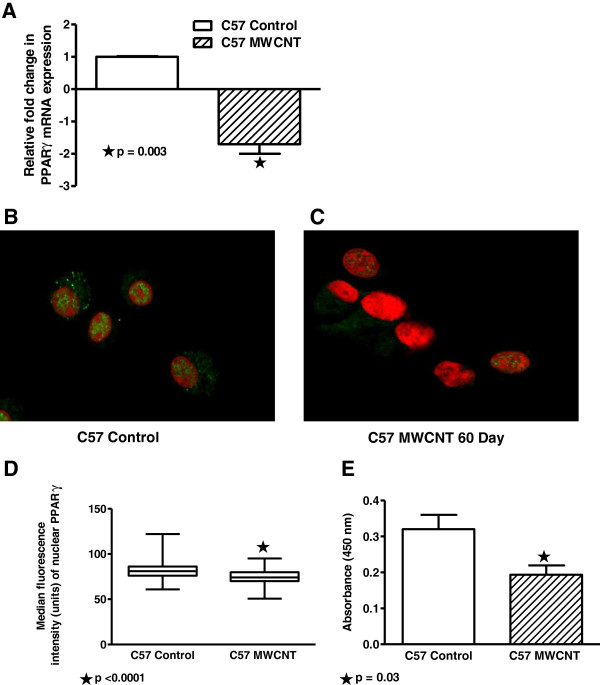
**PPARγ expression and activity are decreased in BAL from MWCNT-instilled wild-type C57Bl/6 mice. **(**A**) BAL cell mRNA samples from MWCNT or sham-treated mice (n=4/group) were analyzed by quantitative PCR. PPARγ mRNA was significantly decreased (p = 0.003) 60 days post MWCNT instillation. Immunocytochemistry of alveolar macrophages collected by cytospins demonstrated decreased nuclear staining for PPARγ protein at 60 days after MWCNT instillation (**C**) compared to sham controls (**B**). (Nuclei were counterstained with propidium iodide). (**D**) Quantification of PPARγ fluorescence intensity of alveolar macrophages in immunostained cytospins (n=99 cells/slide) indicated MWCNT exposure significantly reduced PPARγ expression (n=5/group). (**E**) PPARγ binding activity was reduced in alveolar macrophages of MWCNT-treated mice (n=3/group) as measured by ELISA.

Analysis of mRNA derived from BAL cells revealed significant reduction of PPARγ mRNA expression in wild-type mice at 60 days after MWCNT (100 ug) instillation as compared to sham controls (Figure 1A). Direct examination of alveolar macrophage PPARγ expression by immunocytochemistry confirmed reduction of PPARγ protein at 60 days of MWCNT exposure (Figures 1B-D). Similarly, PPARγ DNA-binding activity also decreased (Figure 1E). Thus all parameters indicated that MWCNT instillation into the lung induced severe and significant repression of alveolar macrophage PPARγ expression and functional activity at 60 days of exposure.

### Granuloma formation in macrophage-specific PPARγ KO mice exceeds that of wild-type animals

**Figure 2:**
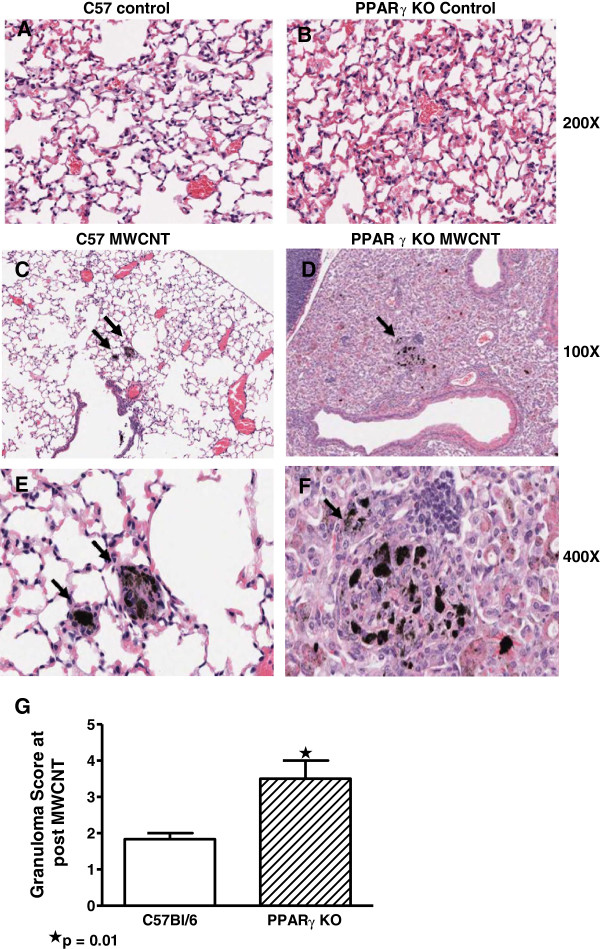
**Extent of granuloma involvement is greater in PPARγ KO than in wild-type lung tissues.** Control lung sections were derived from sham treated wild-type C57/Bl6 (**A**) and (**B**) sham treated PPARγ KO mice (200X). (**C**) Lung sections from C57Bl/6 mice at 60 days after MWCNT instillation show a few aggregates of cells resembling macrophages visible at 100X. (**E**) At higher power, the aggregates of C57Bl/6 macrophages do not appear to have coalesced into a recognizable granuloma, with the individual cytoplasmic borders of the macrophages easily seen (400X). (**D**) In contrast, lungs from the PPARγ KO mice demonstrate numerous granulomas containing relatively large aggregates of MWCNT (100X). (**F**) At higher power, the PPARγ KO macrophages appear to have coalesced into a granuloma, with the syncytial-like loss of cytoplasmic borders characteristic of a well-formed granuloma (400X). (**G**) Analysis by scoring index indicates increased pulmonary granuloma size (p = 0.01) in PPARγ KO compared to wild-type C57Bl/6 mice (n=6/group).

To examine the effects of pre-existing PPARγ deficiency on MWCNT granuloma formation, MWCNT (100 μg) were instilled into lungs of macrophage-specific PPARγ KO and into wild-type mice. For reference, histologic sections from untreated C57/Bl/6 (Figure 2A) and PPARγ KO (Figure 2B) are provided. Granuloma formation was evaluated at 60 days of MWCNT exposure. Histologic comparison of wild-type C57Bl/6 (Figure 2C and E) and PPARγ KO (Figure 2D and F) lung tissues was carried out using a simplified scoring system taking into account size and frequency of granulomas. Results indicated that the extent of granuloma formation in PPARγ KO mice was significantly (p = 0.01) greater than in wild-type mice (Figure 2E).

### Recruitment of CD3+ T cells and macrophages

**Figure 3:**
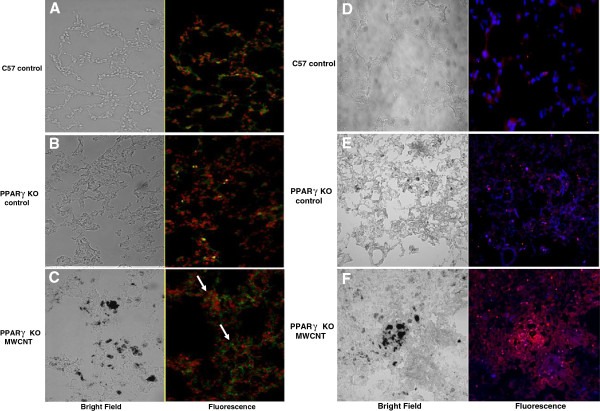
**Recruitment of CD3+ T Cells and Macrophages.** Bright field images of each section are shown in the left panel and corresponding fluorescent images are on the right**.** Images of control lung sections from sham-treated C57/Bl6 (**A**) and PPARγ KO (**B**) mice stained with anti-CD3 demonstrate minimal CD3+ staining. In contrast, lung tissue sections from MWCNT-instilled PPARγ KO mice exhibit marked CD3+ staining in association with granulomatous foci (**C**). Minimal MOMA staining is found in control lung tissue sections from sham treated C57Bl/6 (**D**) and PPARγ KO (**E**) mice. In MWCNT-instilled PPARγ KO mice, lung sections show prominent MOMA staining localizing with granulomatous foci (**F**).

In order to identify cell types associated with granulomatous tissue, lung sections from nanotube-instilled mice were stained with anti-CD3 (T cells) and anti-MOMA (monocytes and macrophages). Figures 3A and B show minimal CD3+ staining in tissue sections from sham treated C57/Bl6 or PPARγ KO control mice. In contrast, marked CD3+ staining is apparent in association with granulomas in nanotube-instilled mice (Figure 3C). Similarly, Figures 3D and E demonstrate minimal MOMA staining in lung tissues from sham treated mice but prominent MOMA staining localizing with granulomatous foci in nanotube-instilled mice (Figure 3F).

### Pro-inflammatory cytokines are elevated in granulomatous tissue

**Figure 4:**
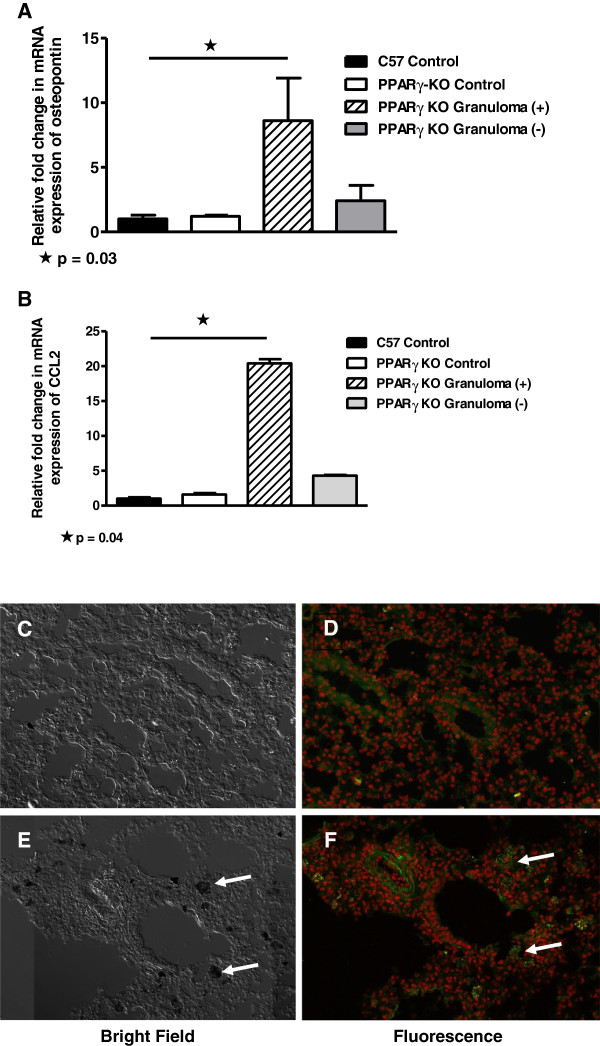
**Granulomatous tissues contain elevated pro-inflammatory cytokine expression in macrophage-specific PPARγ KO mice.** QPCR of PPARγ KO lung tissues obtained by laser capture microdissection reveal elevated mRNA expression of osteopontin (**A**) (p = 0.03) and CCL2 (**B**) (p = 0.04) in granulomatous foci (MWCNT) compared to control wild type lung tissues (n=2/group). Unstained PPARγ KO lung tissue sections (200x) were obtained from control lung (**C**) or MWCNT-instilled lung (**E**). Arrows point to MWCNT within granulomas (**E**). Additional lung tissue sections were stained with antibody to IFN-γ and counterstained with propidium iodide to localize nuclei: (**D**) Sham control lung; (**F**) MWCNT-instilled lung. Arrows point to IFN-γ -positive cells within granulomatous foci (**F**).

BAL cell counts were not affected by MWCNT exposure (data not shown), however, as we noted previously, lymphocyte counts from untreated PPARγ KO mice (7.6 ± 1.4%) were significantly (p = 0.005) higher than those of wild-type (3.2 ± 0.6%) [16]. Examination of lung tissues by laser microdissection and qPCR revealed that mRNA expression of osteopontin, a granuloma-promoting chemokine [17], was elevated in granulomatous foci compared to wild type sham control lung tissues (Figure 4A). Previously in wild-type mice we found that osteopontin was elevated 20-fold in granulomatous foci at 60 days whereas non-granulomatous tissue did not differ from untreated lung tissue [12]. Elevated expression of the monocyte chemokine, CCL2 [18] was also prominent in granuloma foci compared to control wild type tissue (Figure 4B). CCL2 expression in granulomas was also investigated previously in wild-type mice and found to be elevated 6-fold compared with lung tissue from controls [12]. Although elevation of mRNA from the prototypical inflammatory cytokine, interferon gamma (IFN-γ), was below detectable limits in LCM samples of granulomatous foci, immunostaining confirmed the presence of numerous IFN-γ-expressing cells within 60-day MWCNT-induced granulomas (Figures 4E and F). In contrast, sham control lung tissue contained few such cells (Figures 4C and D).

### Pro-inflammatory Cytokines are elevated in BAL cells and fluids from MWCNT-instilled PPARγ KO mice

**Figure 5:**
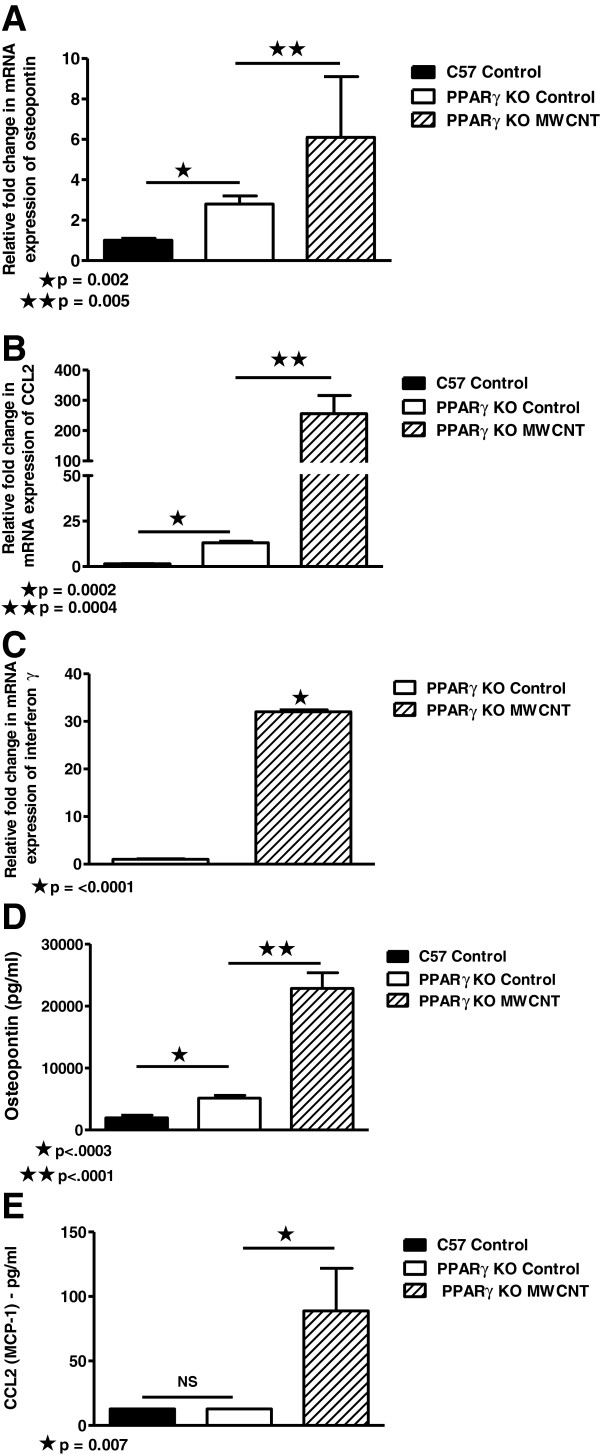
**Pro-inflammatory cytokines are elevated in BAL cells and fluids from macrophage-specific PPARγ KO mice 60 days post-instillation of MWCNT. **(**A**) MWCNT instillation of PPARγ KO mice (n=8) significantly increases BAL cell osteopontin mRNA expression compared to sham C57/Bl6 (n=7) or PPARγ KO (n=6) controls. (**B**). CCL2 mRNA expression is increased in MWCNT-instilled PPARγ KO mice (n=11) compared to sham C57/Bl6 (n=9) or PPARγ KO (n=8) controls. (**C**) IFNγ mRNA expression is increased in MWCNT-instilled PPARγ KO (n=11) compared to PPARγ KO controls (n=8). BAL fluids from MWCNT-exposed PPARγ KO mice contain elevated: (**D**) osteopontin compared to sham C57Bl/6 or PPARγ KO controls (n=8/group); and (**E**) CCL2 (n=6/group) proteins compared to sham controls. CCL2 levels of C57Bl/6 and PPARγ KO sham control groups were below detectable limits of the assay (12.8 pg/ml).

Because previous findings in BAL cells from wild-type mice indicated elevation of pro-inflammatory cytokines at 60 days after MWCNT instillation [12], we investigated BAL cells in macrophage-specific PPARγ KO mice. Sixty days after MWCNT instillation, PPARγ KO BAL cells contained elevated mRNA expression levels of osteopontin (Figure 5A) and CCL2 (Figure 5B) compared to sham C57/Bl6 or PPARγ KO controls. IFN-γ mRNA was also elevated in nanotube-instilled PPARγ KO compared to sham control PPARγ KO (Figure 5C). Previously, we had reported that IFN-γ mRNA was elevated in untreated PPARγ KO mice compared to untreated wild-type C57Bl/6 [13]. Analysis of BAL fluids also demonstrated elevated osteopontin (Figure 5D) and CCL2 (Figure 5E) proteins at 60 days post-MWCNT instillation. Although IFN-γ protein was apparent within granulomas (Figure 4F), IFN-γ was below detectable limits in BAL fluids.

## Discussion

Data from the present study indicate that both expression and activity of PPARγ in alveolar macrophages from wild-type mice were significantly diminished at 60 days after pulmonary instillation of nanotubes. These data supported previous findings of reduced PPARγ in alveolar macrophages from patients with the human chronic granulomatous disease, sarcoidosis [15]. Investigation of the effects of pre-existing PPARγ deficiency was achieved by utilizing a conditional mouse model in which PPARγ was specifically disrupted in macrophages and neutrophils [16]. Results from this model revealed elevated pro-inflammatory cytokines in granulomatous tissue, BAL cells, and BAL fluids 60 days post-instillation of MWCNT. Histological examination of lung tissues from MWCNT-exposed animals also indicated a greater extent of granuloma formation in PPARγ KO mice than in MWCNT-exposed wild-type animals.

The stimulatory effects of MWCNT instillation on cytokine production were noted previously in wild-type C57Bl/6 mice [12]. The current report also indicates that MWCNTs repress PPARγ expression in wild-type mice, raising the question of whether PPARγ deficiency augments pro-inflammatory cytokine expression. Previously, we reported that IFN-γ and other Th1-type pro-inflammatory cytokines/chemokines were intrinsically elevated in untreated PPARγ KO mice compared to wild-type mice [16]. This increased cytokine expression was reduced by *in vivo* administration of a PPARγ lentivirus construct [16]. Additionally, application of a PPARγ antagonist to healthy wild-type alveolar macrophages *in vitro* resulted in an elevated cytokine profile resembling the PPARγ KO phenotype [16]. When taken together, current and previous data allows us to hypothesize that healthy alveolar macrophage PPARγ expression is necessary to restrain pro-inflammatory cytokine expression and maintain pulmonary integrity.

Despite the presence of elevated intrinsic cytokines in PPARγ KO mice compared to wild-type controls, MWCNT instillation further elevated cytokine expression when compared to sham PPARγ KO controls. Cytokines elevated by MWCNT exposure in PPARγ KO mice included osteopontin, which was found in granuloma tissue, BAL cells and fluids. Osteopontin is a noncollagenous matrix protein with cytokine properties that include cellular activation, migration and cell-matrix interaction in T lymphocytes, macrophages, and fibroblasts [17,19]. Osteopontin is abundant in granulomas of varying etiology [20] and deficiency of this molecule in null mice impairs granuloma formation [17]. Gene expression of osteopontin is antagonized by PPARγ ligands, thus suggesting a direct avenue by which PPARγ may attenuate granuloma formation [21].

Interestingly, osteopontin is reported to increase T cell expression of IFN-γ [22] and IFN-γ has been shown to induce osteopontin in monocyte-derived macrophages [23]. Thus it is possible that IFN-γ and osteopontin may interact in a positive feedback loop to maintain high levels in untreated PPARγ KO mice. In human sarcoidosis, both osteopontin and IFN-γ are prominent in granulomas [24,25] and IFN-γ is persistently elevated in sarcoidosis BAL cells [26-28].

BAL cells from untreated PPARγ KO mice also exhibited higher intrinsic mRNA levels of CCL2 (MCP-1), a potent monocyte chemokine [18] than did BAL cells from wild-type mice. Previously we noted that CCL2 expression in wild-type C57Bl/6 mice became elevated at sixty days post-instillation of MWCNT [12]. CCL2 was also further elevated in PPARγ KO BAL cells at 60 days post MWCNT instillation. Interestingly, elevated CCL2 is also present in sarcoidosis BAL cells and fluids [29] as well as serum [30]. Overall, the evidence for elevated CCL2 in human sarcoidosis and in MWCNT-instilled wild-type and PPARγ KO mice suggests that this chemokine is an important element of inflammatory pulmonary granuloma formation.

PPARγ plays an important role in the negative regulation of inflammation by inhibiting the gene expression of numerous cytokines including osteopontin and CCL2. Reported mechanisms of PPARγ-mediated transrepression include: (a) binding of NF-κB, (b) induction of IκBα expression (c) inhibition of MAPK activity, (d) competition for coactivators (e.g. CREB-binding proteins), and (e) blocking clearance of NCoR corepressor complexes [31]. Thus in the current study, we hypothesize that a lack of PPARγ-mediated transrepression may be responsible for augmenting granulomatous reactions in macrophage-specific PPARγ KO mice via concurrent upregulation of cytokines (osteopontin and CCL2) involved in granuloma formation.

## Conclusion

Chronic granulomatous inflammation in response to chemical or biological insult has proven to be a difficult disease to control, despite years of research. Nanoparticles have only recently been implicated as potential causes of granuloma formation that may relate to either occupational or environmental exposures [32]. The persistent nature of the MWCNT granuloma model parallels that of human granulomatous disorders and emphasizes the applicability of this model to studies of human granuloma pathophysiology. The current findings provide a new perspective on the critical role of alveolar macrophage PPARγ in lung homeostasis by demonstrating negative effects on granuloma formation.

## Competing interests

The authors have no financial competing interest.

## Authors’ contribution

IH contributed to acquisition of the data, analysis and interpretation of data, drafting of the manuscript and final approval of the version to be published. AM contributed to acquisition of the data, analysis and interpretation of data, drafting of the manuscript and final approval of the version to be published. JP contributed to the acquisition of data and final approval of the version to be published. MM contributed to the acquisition of data and final approval of the version to be published. LD contributed to the acquisition of data and final approval of the version to be published. CW contributed to the conception and characterization/preparation of nanotubes. BPB contributed to the design, analysis and interpretation of data, drafting of the manuscript and final approval of the version to be published. MJT contributed to the design, analysis and interpretation of data, drafting of the manuscript and final approval of the version to be published. All authors read and approved the final manuscript.
